# Antibody-Mediated Rejection of Arterialised Venous Allografts Is Inhibited by Immunosuppression in Rats

**DOI:** 10.1371/journal.pone.0091212

**Published:** 2014-03-11

**Authors:** Katrin Splith, Peter Fellmer, Ivan Matia, Martin Varga, Martin Oliverius, Stephanie Kuhn, Linda Feldbrügge, Felix Krenzien, Hans-Michael Hau, Georg Wiltberger, Moritz Schmelzle, Sven Jonas

**Affiliations:** 1 Translational Centre for Regenerative Medicine, University of Leipzig, Leipzig, Germany; 2 Department of Visceral, Transplantation, Thoracic and Vascular Surgery, University of Leipzig, Leipzig, Germany; 3 Transplant Surgery Department, Institute for Clinical und Experimental Medicine, Prague, Czech Republic; Xavier Bichat Medical School, INSERM-CNRS - Université Paris Diderot, France

## Abstract

**Objectives and Design:**

We determined in a rat model (1) the presence and dynamics of alloantibodies recognizing MHC complexes on quiescent Brown-Norway (BN) splenic cells in the sera of Lewis (LEW) recipients of Brown-Norway iliolumbar vein grafts under tacrolimus immunosuppression; and (2) the presence of immunoglobulins in the wall of acute rejected vein allografts.

**Materials and Methods:**

Flow cytometry was used for the analysis of day 0, 14 and 30 sera obtained from Lewis recipients of isogeneic iliolumbar vein grafts (group A) or Brown-Norway grafts (group B, C) for the presence of donor specific anti-MHC class I and II antibodies. Tacrolimus 0.2 mg/kg daily was administered from day 1 to day 30 (group C). Histology was performed on day 30.

**Results:**

Sera obtained preoperatively and on day 30 were compared in all groups. The statistically significant decrease of anti MHC class I and II antibody binding was observed only in allogenic non-immunosuppressed group B (splenocytes: MHC class I - day 0 (93%±7% ) vs day 30 (66%±7%), *p* = 0.02, MHC class II - day 0 (105%±3% ) vs day 30 (83%±5%), *p* = 0.003; B-cells: MHC class I - day 0 (83%±5%) vs day 30 (55%±6%), *p* = 0.003, MHC class II - day 0 (101%±1%) vs day 30 (79%±6%), *p* = 0.006; T-cells: MHC class I - day 0 (71%±7%) vs day 30 (49%±5%), *p* = 0.04). No free clusters of immunoglobulin G deposition were detected in any experimental group.

**Conclusion:**

Arterialized venous allografts induce strong donor-specific anti-MHC class I and anti-MHC class II antibody production with subsequent immune-mediated destruction of these allografts with no evidence of immunoglobulin G deposition. Low-dose tacrolimus suppress the donor-specific antibody production.

## Introduction

There remain a group of vascular patients with critical leg ischemia who are not suitable for the use of greater saphenous vein or prosthetic grafts in peripheral vascular reconstruction. In specific indications, allogeneic veins are used in these patients.[1] However, allogeneic veins are immunogenic because of the expression of both class I and class II major histocompatibility complex (MHC) antigens on their wall cells.[2] These antigens stimulate immune responses in the host that lead to the destruction of the allovenous wall structure. The rejection is represented by graft thrombosis or by graft dilatation with the risk for graft rupture.[3], [4]

One possibility for increasing the patency rates of venous allografts is the use of immunosuppressive drugs.[5] However, immunosuppression is not routinely used in clinical practice.[1] When immunosuppression is used, cyclosporine A is the most frequently administered drug to patients with allovenous bypasses.[6]–[8].

However, recently published data confirmed considerable vascular side effects from cyclosporine A.[9], [10] Contrary to cyclosporine A, tacrolimus, a newer and more potent immunosuppressive drug routinely used in renal and liver transplant patients, showed significantly advantageous characteristics related to hypertension, dyslipidaemia, and renal function in transplant patients.[9] Moreover, tacrolimus seems to be a promising compound in a new generation of coronary drug eluting stents.[11].

In our previous experiments, we used the rat ileolumbar vein to abdominal aorta transplantation model to study the effect of low-dose tacrolimus immunosuppression on rejection changes and adaptation of venous allografts to arterialisation in rats. Tacrolimus inhibited cell-mediated rejection, and the immunosuppressed alloveins developed characteristic signs of the wall remodelling process observed in syngeneic arterialised veins.[12].

However, the significance of antibody-mediated rejection in chronic vascular rejection and consecutive failure of transplanted organs seems to be more and more important.[13] Moreover, the production of donor-specific antibodies against the major histocompatibility complex (anti-MHC) in dogs was clearly connected with venous allografts thrombosis.[4].

In the present study, we determined the following parameters: (1) the presence and dynamics of alloantibodies recognizing MHC complexes on Brown-Norway (BN) splenocytes, quiescent BN splenic B-cells and T-cells in the sera of Lewis (LEW) recipients of BN iliolumbar vein grafts under low dose tacrolimus immunosuppression; and (2) the presence of immunoglobulin in the rejected allovein wall. For this purpose, we screened for the presence of donor-specific anti-MHC class I and II antibodies in recipient’s sera that was obtained previously in our Brown-Norway to Lewis rat model of allovenous arterialisation.[12].

## Materials and Methods

### Ethics statement

The principles of laboratory animal care were followed and all rats were maintained according to the National Institute of Health Guidelines. Ethical approval by a local ethical committee of the Institute for Clinical and Experimental Medicine was obtained for this study.

### Animals

Adult male inbred Brown-Norway (BN; RT1^n^) and Lewis (LEW; RT1^l^) rats were obtained from Charles River (Sulzfeld, Germany). Male LEW rats (N = 23, 200–340 g) were used as recipients of allogeneic or syngeneic iliolumbar vein grafts. Male BN rats (N = 9, 220–300 g) were used as donors of allogeneic iliolumbar vein grafts. Male LEW rats (N = 3, 280–300 g) were used as donors of syngeneic iliolumbar vein grafts. Each transplanted animal was housed in a separate cage during the entire 30-day follow-up period. Only animals that survived the whole follow-up period were included in this study.

### Operative procedure

Iliolumbar vein transplantation into the abdominal aorta was described in detail in our previous publication.[12] Briefly, donor animals were anaesthetised with an intramuscular injection of ketamine 100 mg/kg (Narkamon, Spofa a.s., Prague, Czech Republic) and xylazine 10 mg/kg (Rometar, Spofa a.s., Prague, Czech Republic). Two segments of the iliolumbar veins (1–1.5 cm in length) were excised and stored in saline solution at room temperature until transplantation. The median ischemic time for the vein allografts was 160 min.

The recipient animals with planned follow-up were anaesthetised with less invasive anaesthesia (intramuscular injection of 20 µg/kg sufentanil (Sufenta, Janssen Pharmaceutica Inc., Beerse, Belgium) and 1 mg/kg azaperone (Stresnil, Janssen Pharmaceuticals Inc., Beerse, Belgium) to ensure a more natural awakening.

The venous allografts were implanted into the infrarenal aorta of the recipient rats after a midline laparotomy using a running 10/0 monofilament suture (Ethicon Inc., Sommerville, New Jersey, USA).

Neither anticoagulants nor anti-platelet drugs were used in the experiment.

### Animal groups

The recipient animals were divided into three groups. Animals in group A received isogeneic venous transplantation (LEW to LEW, N = 4), and animals in group B received allogeneic venous transplantation (BN to LEW, N = 9). Neither group received immunosuppressive therapy. Animals in group C received allogeneic venous transplantation (BN to LEW, N = 7) and were immunosuppressed with daily intramuscular injections of low-dose tacrolimus (0.2 mg/kg daily).

### Immunosuppressive therapy

The immunosuppressive protocol of allogeneic group C animals was the same as described previously.[12] Briefly, tacrolimus (Prograf, Astellas Pharma Inc., Tokyo, Japan) was suspended in saline solution and administered intramuscularly in daily doses of 0.2 mg/kg. Immunosuppression was initiated on day 1 after transplantation and was administered for the entire 30-day follow-up period. On day 30, tacrolimus blood levels were evaluated using an enzyme-enhanced immunoassay technique (Emit 2000 Tacrolimus assay, Dade Behring Inc., Deerfield, Illinois, USA), and the venous grafts were removed after a midline re-laparotomy and processed for histology and immunohistochemistry (see below). The animals were then euthanized by intracaval administration of a lethal dose of thiopental (Thiopental, Spofa a.s., Prague, Czech Rep).

### Blood samples

Blood samples were collected in all groups on days 0, 14, and 30 by orbital sinus puncture as described by van Herck.[14]

### Splenocytes

Spleens from other male BN rats (N = 10, weight 200–250 g, Charles River, Sulzfeld, Germany) were used as a source of splenocytes for this study. The spleens were removed after midline laparotomy in anesthetized animals (intramuscular anaesthesia with sufentanil and stresnil as described above). The animals were then killed with intracaval administration of a lethal dose of thiopental (Thiopental, Spofa, Czech Republic). The spleens were removed and immediately processed according to a protocol for splenocyte preparation used in our laboratory. Briefly, the excised spleen was minced into small pieces, pressed through a strainer using the plunger end of a syringe, and washed with phosphate buffered saline solution (PBS). The cell suspension was placed on Biocoll separating solution (BioScience, Nottingham, UK) and centrifuged at 2000 rpm for 20 minutes. The supernatant was resuspended in 10% foetal calf serum (FCS) with RPMI 1640 (Sigma-Aldrich Chemie GmbH, Buchs SG, Switzerland) and centrifuged at 1200 rpm for 10 minutes. This step was done twice. A cell count and viability check using trypan blue was performed afterwards. Cells were then stored in tubes with freezing medium (Iscove’s Modified Dulbecco’s Medium + 20% FCS) in liquid nitrogen until processing.

### Flow cytometry analysis

*In vitro* binding of sera obtained in all three animal groups and quiescent BN splenocytes was determined by flow cytometry as described previously.[15] Briefly, cells were thawed, washed in phosphate-buffered saline (PBS), and resuspended in PBS solution with 1% foetal bovine serum (FBS). One hundred thousand cells were incubated for 30 min at 4°C with 10 µl of rat serum. Cells were washed twice in PBS (1% FBS) then incubated with original antibodies as follows: MHC expression on quiescent BN splenocytes was determined using a Biotin-MHC class I (anti-RT1.Ac, OX-27, Acris Antibodies GmbH, Herford, Germany) or a Biotin-MHC class II (anti-RT1.D, OX-17, BD Biosciences, Heidelberg, Germany) primary antibody and a PE-Cy7-streptavidin secondary antibody (BD Biosciences, Heidelberg, Germany). Furthermore, spleen cells were incubated with PE-CD3 (anti-CD3, G 4.18, BD Biosciences, Heidelberg, Germany) and stained with FITC-CD45RA antibody (anti-CD45, OX 33, BD Biosciences, Heidelberg, Germany) to distinguish between T- and B-cells. Ten thousand cells were acquired on a FACSCanto II flow cytometer (BD Biosciences, Heidelberg, Germany) and analysed using FACSDiva software (BD Biosciences, Heidelberg, Germany). Graphic presentations as histograms allowed the determination of mean fluorescence intensity on a log scale.

MHC class I or class II antibody binding of the cells without previous serum incubation was set to 100%.

### Detection of immunoglobulins in the venous wall

Immunohistochemical analysis of transplanted iliolumbar veins was performed according to methods described previously.[12] Briefly, after removal the veins were embedded in Sakura Finetek Tissue Tek Cryomold holders (Sakura Finetek, Tokyo, Japan) and Sakura Finetek Tissue Tek O.C.T. compound (Sakura Finetek, Tokyo, Japan). The samples were frozen in 2-methylbutane (Fluka Chemika, Buchs, Switzerland), cooled with liquid nitrogen, and stored until processed at −80°C. After processing, the 8-µm thick sections were rinsed in PBS and air-dried. The tissues were then incubated with an antibody directly conjugated with fluorescein isothiocyanate (Chemicon International Inc, Temecula, California, USA) for 30 min. The specimens were then dipped in glycerine medium and immediately analysed under a fluorescence microscope.

### Statistical analysis

Values are expressed as the mean ± standard error measurement (SEM). Comparisons between two groups were made using Student’s t-test. Values of *p*<0.05 were considered statistically significant.

## Results

The results of the transplantation, histology, immunohistochemistry, and cell-mediated rejection of iliolumbar vein grafts were presented in detail previously.[12] Immunosuppressive therapy with tacrolimus was necessary for the adaptation of the venous allograft to arterialisation in the previous study.[12]

In the present study, we determined the following parameters: (1) the presence and dynamics of alloantibodies recognizing MHC complexes on quiescent BN splenic B-cells and T-cells in the sera of LEW recipients of BN iliolumbar vein grafts using different fluorescence-labelled antibodies; and (2) the presence of immunoglobulin in the venous wall. The serum antibodies from allografted LEW rats, where presented, were competitive binding to MHC class I and MHC class II molecules on splenocytes and quiescent splenic BN B-cells and T-cells. The inhibition of the fluorescence-labelled MHC class I and II antibody binding consequently decreased the measured fluorescence signal.

### MHC class I positive splenic cells

**Figure 1:**
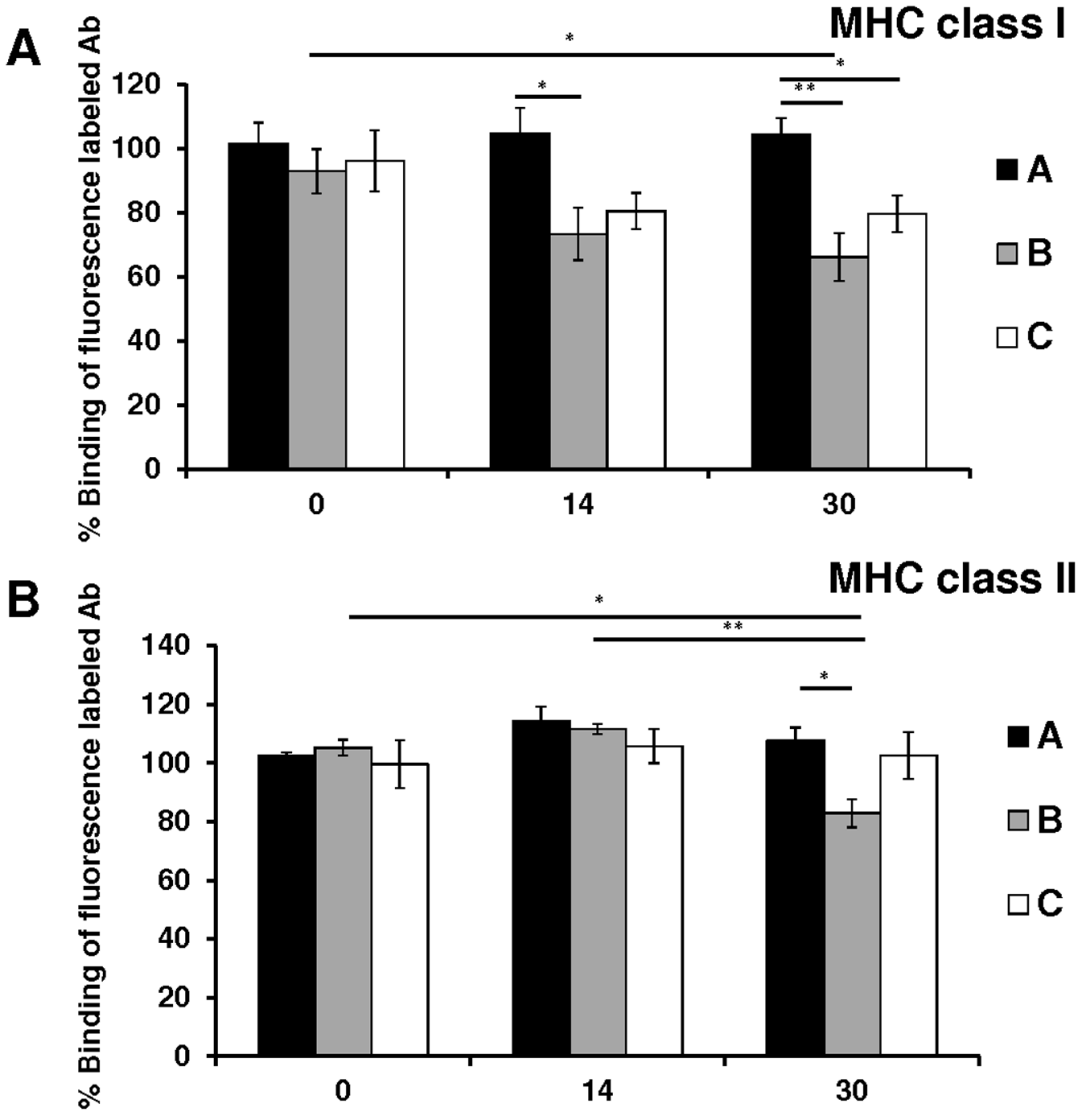
Dynamic of anti splenic cells MHC class I and II antibodies concentrations. The percentage binding of the fluorescence-labelled MHC class I **(A)** and MHC class II **(B)** antibody to BN splenic cells in the presence of sera from group A, B, or C obtained on day 0, 14, or 30 after transplantation. Sera from the allogeneic non-immunosuppressed group B, taken on day 30, significantly inhibited fluorescence-labelled MHC class I and MHC class II antibody binding to splenic cells, compared to day 0 sera. Error bars represent SEM, *p<0.05, **p<0.01.

Blood samples were collected preoperatively (day 0) and on day 14 and 30 after transplantation. Syngeneic group A sera showed no inhibition of the fluorescence-labeled MHC class I antibody binding to BN-splenocyte during the entire follow-up period. (Fig. 1A).

By contrast, sera from allogeneic non-immunosuppressed group B animals obtained on day 30 after transplantation significantly decreased the binding of fluorescence-labeled MHC class I antibody to BN spleen cells (66%±7%), compared with day 0 sera (93%±7%, *p* = 0.02).

In addition, sera from the allogeneic non-immunosuppressed group B obtained on day 14 and day 30 showed significant inhibition of fluorescence-labelled MHC class I antibody binding to BN spleen cells (73%±8%; 66%±7%), compared with day 14 and day 30 sera from the syngeneic group A (104%±8%, *p* = 0.03; 104%±5%, *p* = 0.002).

Allogeneic immunosuppressed group C sera obtained on day 30 showed no significant inhibition of fluorescence-labeled MHC class I antibody binding (79%±6%) compared with day 0 sera (96%± 7%). Compared to group A sera (104%±5%) showed group C day 30 sera significant inhibition (79%±6%, *p* = 0.02).

### MHC class II positive splenic cells

Syngeneic group A sera as well as allogeneic immunosuppressed group C sera showed no significant inhibition of the fluorescence-labeled MHC class II antibody binding to BN spleen cells during the entire follow-up period (Fig.1B ).

By contrast, day 30 sera from the allogeneic non-immunosuppressed group B rats showed significant inhibition of fluorescence-labelled MHC class II antibody binding to BN spleen cells (83%±5%) compared with group B day 0 sera (105%±3%, *p* = 0.003) and day 14 sera (112%±2%, *p* = 0.002) as well as day 30 sera from the syngeneic group A (108%±5%, *p* = 0.006).

### MHC class I positive splenic B-cells

Quiescent BN splenic B-cells were identified as CD45RA-positive cells.

**Figure 2:**
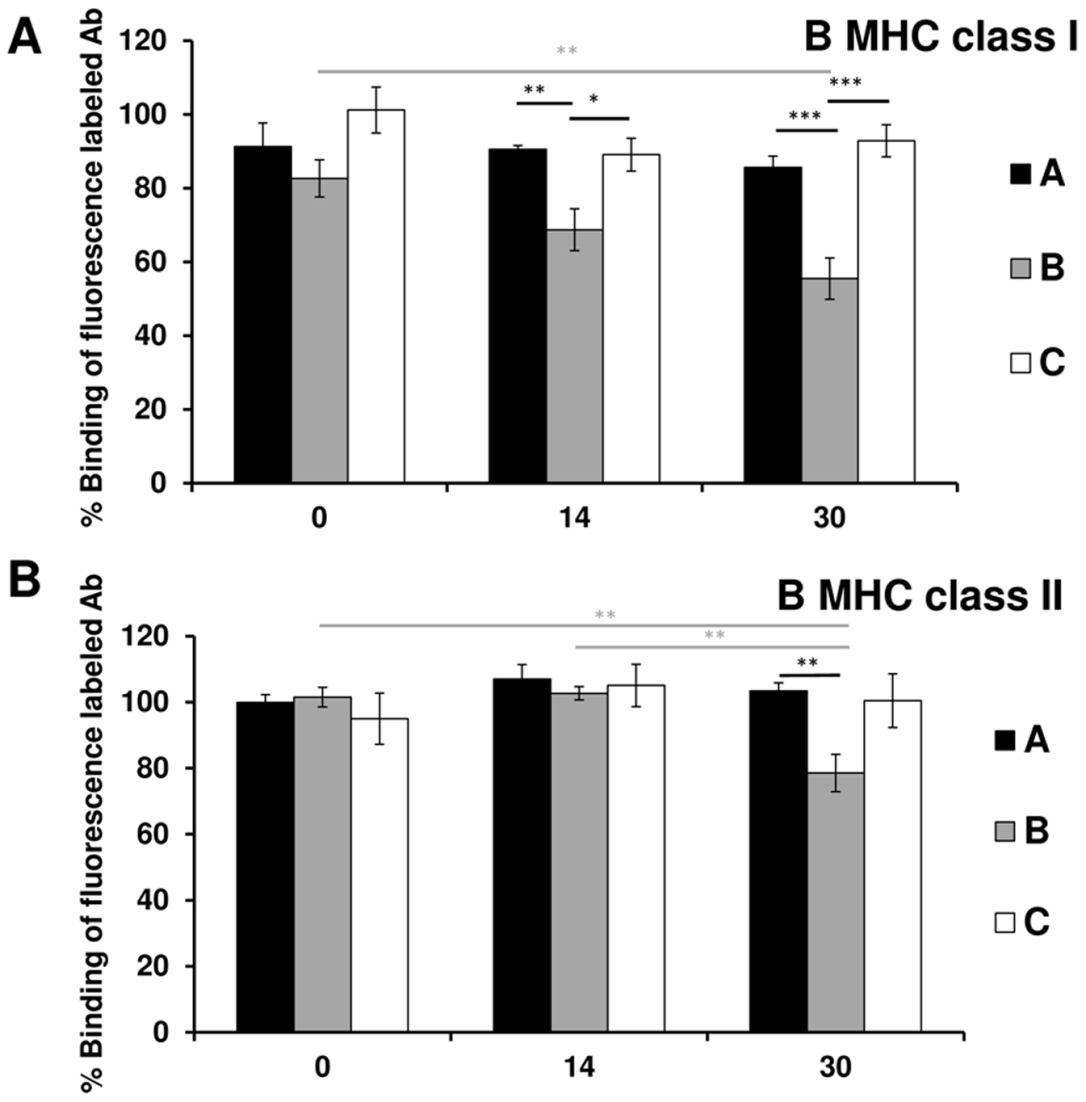
Dynamic of anti splenic B-cells MHC class I and II antibodies concentrations. The percentage binding of the fluorescence-labelled MHC class I **(A)** and MHC class II **(B)** antibody to BN splenic B-cells, identified as CD45RA-positive cells, in the presence of sera from group A, B, or C obtained on day 0, 14, or 30 after transplantation. Only sera from the allogeneic non-immunosuppressed group B, taken on day 14 or day 30, significantly inhibited fluorescence-labelled MHC class I and MHC class II antibody binding to splenic B-cells. Error bars represent SEM, *p<0.05, **p<0.01, ***p<0.001.

Syngeneic group A sera as well as allogeneic immunosuppressed group C sera showed no significant inhibition of fluorescence-labelled MHC class I antibody binding to BN B-cells during the entire follow-up period (Fig. 2A).

By contrast, sera from allogeneic non-immunosuppressed group B obtained on day 14 and day 30 showed inhibition of fluorescence-labelled MHC class I antibody binding. This binding was significantly decreased in the presence of day 30 sera (55%±6%) compared with day 0 sera (83%±5%, *p* = 0.003).

In addition, sera from the allogeneic non-immunosuppressed group B obtained on day 14 and day 30 showed significant inhibition of fluorescence-labelled MHC class I antibody binding to splenic B-cells (69%±6%; 55%±6%) compared with day 14 and day 30 sera from the syngeneic group A (91%±1%, *p* = 0.007; 86%±3%, *p* = 0.00007) as well as the day 14 and day 30 sera from allogeneic group C (89%±5%, *p* = 0.02; 93%±4% *p* = 0.0002).

### MHC class II positive splenic B-cells

Quiescent BN splenic B-cells were identified as CD45RA-positive cells.

Sera from the syngeneic group A and allogeneic immunosuppressed group C showed no significant inhibition of fluorescence-labelled MHC class II antibody binding to BN B-cells during the entire follow-up period (Fig. 2B).

By contrast, day 30 sera from the allogeneic non-immunosuppressed group B rats showed significant inhibition of fluorescence-labelled MHC class II antibody binding to B-cells (79%±6%) compared with day 0 (101%±1%, *p* = 0.006) and day 14 (104%±1%. *p* = 0.002) sera.

### MHC class I positive T-cells

Quiescent BN splenic T-cells were identified as CD3-positive cells.

**Figure 3:**
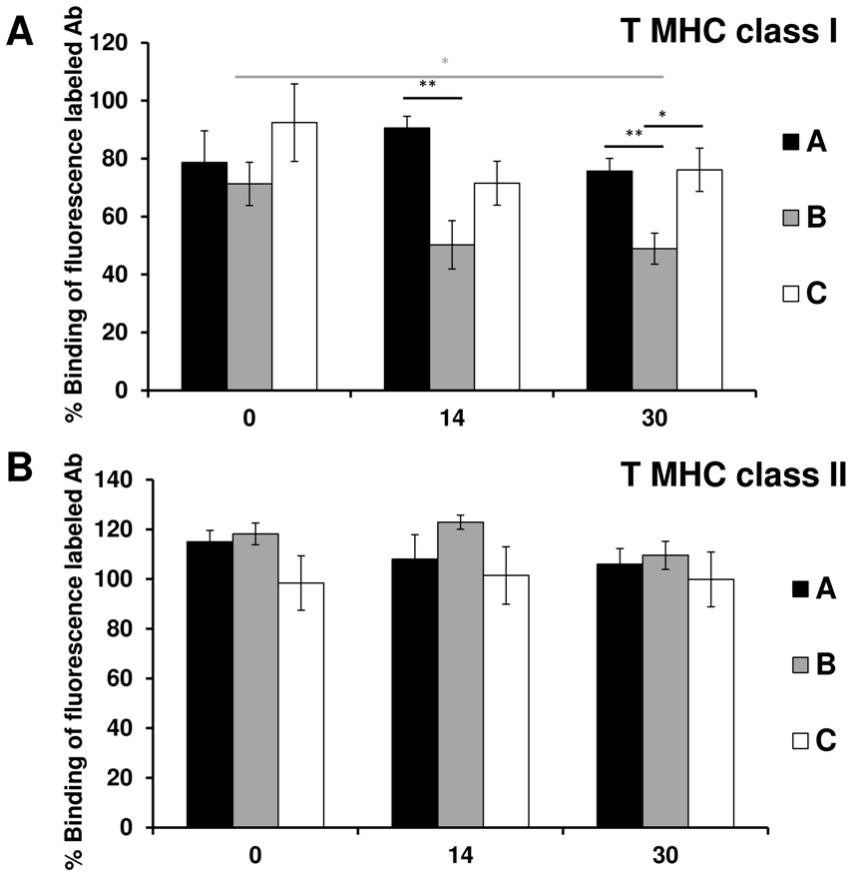
Dynamic of anti splenic T-cells MHC class I and II antibodies concentrations. The percentage binding of the fluorescence-labelled MHC class I **(A)** and MHC class II **(B)** antibody to BN splenic T-cells, identified as CD3-positive cells, in the presence of sera from group A, B, or C obtained on day 0, 14, or 30 after transplantation. Only sera from the allogeneic non-immunosuppressed group B obtained on day 14 or day 30 showed significant inhibition of fluorescence-labelled MHC class I antibody binding to splenic T-cells. Error bars represent SEM, *p<0.05, **p<0.01, ***p<0.001.

No significant inhibition of the fluorescence-labelled MHC class I antibody binding to BN T-cells was observed in sera from syngeneic group A or allogeneic immunosuppressed group C during the entire follow-up period (Fig. 3A).

By contrast, day 30 sera from allogeneic non-immunosuppressed group B showed significant inhibition of fluorescence-labelled MHC class I antibody binding to T-cells (49%±5%) compared with day 0 sera (71%±7%, *p* = 0.04). Additionally, the inhibition observed was significantly stronger compared with syngeneic group A day 30 (76%±4%, *p* = 0.005) and allogeneic immunosuppressed group C day 30 (76%±7%, *p* = 0.02) sera.

### MHC class II positive T-cells

Quiescent BN splenic T-cells were identified as CD3-positive cells. However, these quiescent splenic T-cells do not express MHC class II antigens. We did not observe any significant inhibition of fluorescence-labelled MHC class II antibody binding in the presence of sera obtained from any of the three experimental groups during the entire follow-up period (Fig. 3B).

### Immunoglobulins in the venous wall

**Figure 4:**
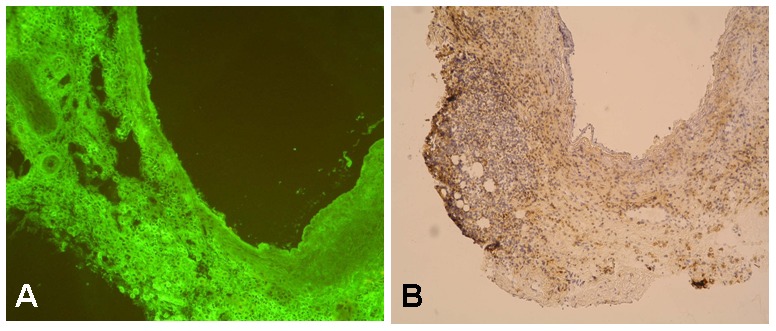
Histological features of rejected non-immunosuppressed alloveins. Representative light microscopic photograph showing the histological features of alloveins in non-immunosuppressed rats (group B) 30 days following transplantation into the infrarenal abdominal aorta: A - vein stained for immunoglobulins with a fluorescein isothiocyanate-conjugated antibody (Chemicon International Inc, Temecula, California, USA). No free clusters of immunoglobulins were detected in the allovenous wall. B - vein stained for CD4+ cells with anti-CD4 antibody (OX-8, Cymbus Biotechnology LTD, Southampton, UK) as described previously.[12] Massive infiltration of CD4+ immunocompetent cells (stained brown) led to the destruction of allovenous wall with no histological signs of arterialisation. Original magnification ×100.

**Figure 5:**
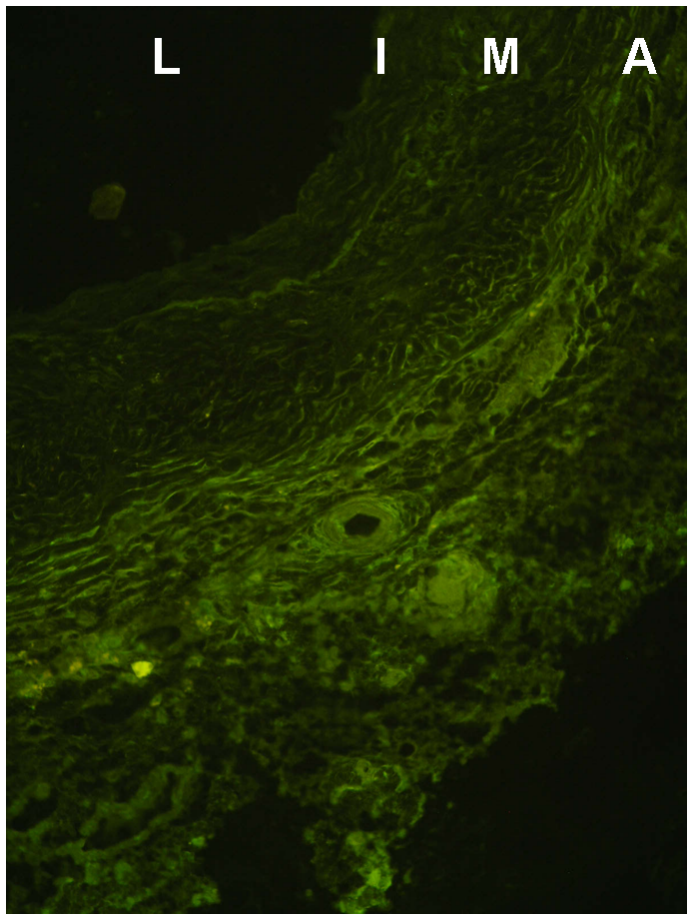
Histological features of arterialised immunosuppressed alloveins. Representative light microscopic photograph showing the histological features of alloveins in immunosuppressed rats with tacrolimus (group B) 30 days following transplantation into the infrarenal abdominal aorta. Vein stained for immunoglobulins with a fluorescein isothiocyanate-conjugated antibody (Chemicon International Inc, Temecula, California, USA). No free clusters of immunoglobulins were detected in the thick arterialised wall of venous allograft. Original magnification ×400. L – lumen, I – tunica intima, M – tunica media, A – tunica adventitia.

Immunofluorescent staining showed no free clusters of IgG deposition on day 30 after arterialisation of both immunosuppressed and non-immunosuppressed iliolumbar alloveins. (Fig.4, Fig. 5) IgG positive fluorescent spots were observed only in the wall of rejected non-immunosuppressed allovenous graft. The area of fluorescence positivity correlated with cellular infiltration of immunocompetent cells.

## Discussion

The results of our experimental study of antibody production after allovenous arterialisation showed massive induction of donor-specific anti-MHC class I and class II antibody production by recipients of allogeneic veins. The allogeneic venous allografts in non immunosuppressed rats did not develop signs of venous wall adaptation to arterialisation. The venous wall, including smooth muscle cells (SMC), was destroyed by a massive infiltration of immunocompetent cells of recipient origin. The SMC were unable to proliferate and adapt to the new biomechanical conditions. However, donor-specific class I and class II antibody production and the destruction of venous allografts were suppressed by low-dose tacrolimus immunosuppression.

The importance of anti-MHC antibody production during the process of rejection of the venous allografts was experimentally documented. Antibody production after histo-incompatible femoral vein to femoral artery interposition in dogs appeared specifically at 4 weeks, and lasted until graft occlusion was detected, between postoperative weeks 4 and 12.[4] Furthermore, 85% of studied animals developed antibodies that activated the complement system and lysed the donor endothelial cells.[16] Inhibiting antibody production in 75% of animals using a combination of cyclosporine A at a dosage of 10 mg/kg per day with mycophenolate mofetil at a dosage of 20 mg/kg per day was observed in animals with a 100% patency rate at 20 weeks. Given alone, neither cyclosporine A nor mycophenolate mofetil improved the overall patency rate of venous allografts, and did not suppress the development of donor-specific antibodies.[16]

The same research group observed the deposition of IgG isotype antibodies in the walls of arterialised venous allografts in dogs 4 to 12 weeks after thrombosis developed.[4] However, the authors were unable to distinguish between real IgG deposition and deposits related to B cell infiltration, as moderate infiltration of mononuclear cells and mild infiltration of plasma cells were observed within the media and adventitia of allografts with thrombosis. In our model, we observed activation of donor-specific anti-MHC class I and class II production during the first 2 weeks after arterialisation. This production was sufficiently suppressed by low-dose tacrolimus immunosuppression, with mean tacrolimus blood levels of 5.6 ng/ml. However, we did not observe any IgG deposition in the walls of rejected venous allografts. The IgG positivity was observed probably only in cell membranes of invading recipient MHC class II positive cells. This is in contrast with the direct involvement of IgG deposition in the destructive process we observed previously in the rejection of non-immunosuppressed arterial allografts.[17] This is probably owing to a greater content of smooth muscle cells and MHC antigens in the arterial wall compared with veins.

The exact role of anti MHC antibodies in the process of venous rejection is not clear. [4] This phenomenon was studied mainly in the process of alloarterial rejection. Thaunat et al. reported in BN to LEW aortic transplant model that anti–MHC I alloantibodies play a key role in the arterial remodeling during the graft rejection.[18], [19] They demonstrated that the binding of anti–MHC class I alloantibodies to the SMCs of the medial donor exerts a sequential biphasic effect. First, they induce a transient production of growth factors that promote an inappropriate response to injury of the intima. These growth factors act in a paracrine fashion to promote the proliferation of SMCs of the recipient that may contribute to the development of an obstructive neointima. In a second phase, the binding of anti–MHC I alloantibodies drives the apoptosis of SMCs of the donor, resulting in the shrinkage of the media.

The importance of anti-MHC antibody production after vessel transplantation in humans was confirmed as well. Strong anti-MHC class I and class II antibody production was observed in the sera of non-immunosuppressed end-stage renal disease patients with an allovenous haemodialysis access.[20] Moreover, the donor-specific anti-MHC class I and class II antibody response was also seen after valved allograft implantation in children with congenital heart disease.[21] This immune-mediated response has the potential for deleterious effects on valved allograft function and persists late after surgery [22] The immunosuppressive therapy with mycophenolic mofetil[21] but not azatioprine [23] reduced this HLA antibody response.

Based on previous reports,[24] cyclosporine A is the immunosuppressant most frequently used by vascular surgeons after venous and arterial allograft implantation over the past 20 years.[6], [25], [26] Balzer et al. were interested in determining the prevalence and specificity of anti-MHC antibodies in vascular patients after peripheral reconstruction with venous allografts.[8] They found a high rate of donor-specific allosensitisation, which included not only a humoral response against constitutively expressed class I antigens, but also extended to class II antigens. This was probably due to upregulation of MHC class II molecules by endothelial cells and SMC in the field of the inflammatory reaction caused by vessel injury, thrombosis, or stasis in the grafted vessels. In this study, all patients received low-dose cyclosporine A immunosuppression with serum levels of 50–90 ng/ml.

Randon et al. reviewed data from patients after cryopreserved vein allograft implantations followed by 1 year long immunosuppressive therapy consisting of cyclosporine A resulting in blood levels of 100 to 150 mg/dL.[6] They concluded that this method led to increased limb salvage and patency rates compared with those described for prosthetic grafts at the infra-popliteal level in most studies. However, no determination of antibody production was performed in these patients.

Mirelli et al. used in 33% of patients after fresh and cryopreserved arterial allografts replacement due to prosthetic graft infection cyclosporine treatment with blood levels between 100 and 200 ng/ml.[25] Despite this treatment, donor-specific anti-MHC class I and class II production was detected. However, antibody production in the cyclosporine A group was less pronounced and was delayed compared with non-immunosuppressed patients.

However, recently published data confirm considerable vascular side effects with use of cyclosporine A.[9], [10] Clinical studies in transplanted patients show that cyclosporine A treatment results in endothelial dysfunction, an important risk factor for cardiovascular adverse events. Moreover, cyclosporine A increased treatments for anti-hypertension and lipid-lowering in these patients [9].

The immunosuppressant tacrolimus and cyclosporine A belong to the group of calcineurin inhibitors. However, tacrolimus is more potent and less toxic compared with cyclosporine A.[27] Experimental arterial transplantation model proved that tacrolimus 0.2 mg/kg is sufficient for survival of endothelial cells of donor origin in arterial allografts; however, cessation of the use of tacrolimus resulted in severe destruction of the arterial wall.[28] The influence of tacrolimus on antibody production was not studied.

Tacrolimus 0.2 mg/kg used in our experiment was established in accordance with the effective dose used in other arterial experiments.[28] This dose led to blood concentrations of 5 ng/ml. Moreover, we confirmed that this low blood concentration inhibits intimal hyperplasia in arterialised syngeneic veins in our previous study,[29] and inhibited histological signs of allogeneic vein rejection [12].

## Conclusions

In conclusion, our data confirm the strong immunogenicity of venous allografts that involve both cell- and antibody-mediated immune responses during the rejection processes of these grafts. In our model, we observed cell-mediated rejection of allografted illiolumbar veins and donor-specific anti-MHC class I and class II antibody production only in non-immunosuppressed allogeneic animals. Signs of cell-mediated rejection were confirmed by massive infiltration and destruction of the venous wall by MHC class II-positive, CD4+, and CD8+ cells of host origin.[12] Moreover, we observed increased donor-specific anti-MHC class I and class II antibody concentrations in the graft recipients sera on days 14 and 30 after transplantation. All these processes were suppressed by low-dose tacrolimus immunosuppression. However, the possible beneficial effect of tacrolimus immunosuppression in vessel allograft implantation in vascular patients need to be confirmed in a clinical study.
